# Decreased erythrocyte nucleoside transport and hENT1 transporter expression in glucose 6-phosphate dehydrogenase deficiency

**DOI:** 10.1186/s12878-015-0038-0

**Published:** 2015-12-19

**Authors:** Mohammad Al-Ansari, James D. Craik

**Affiliations:** Department of Biochemistry, Faculty of Medicine, Health Sciences Center, Kuwait University, PO Box 24923, Safat, 13110 Kuwait

**Keywords:** Glucose-6-phosphate dehydrogenase, Erythrocyte membrane, Nucleoside transporter, Biological transport

## Abstract

**Background:**

Glucose-6-phosphate dehydrogenase (G6PD) deficiency is associated with erythrocyte sensitivity to oxidative damage and hemolytic crises. In β-thalassemia major, where hemoglobin instability imposes oxidative stress, erythrocytes show reduced hENT1 nucleoside transporter expression and decreased nucleoside uptake. This study investigated hENT1 expression and nucleoside transport in G6PD-deficient erythrocytes to determine if decreased hENT1 activity might be a contributory feature in the variable pathology of this enzymopathy.

**Methods:**

Uptake of ^3^H-uridine was measured at room temperature using an inhibitor-oil stop protocol and 5-s incubations. Erythrocyte membranes were analyzed by SDS-PAGE and nucleoside (hENT1), glucose (GLUT-1), and anion exchange (Band 3) transporter polypeptides quantitated on immunoblots.

**Results:**

In G6PD-deficient cells, uridine uptake (mean 8.18, 95 % CI 5.6–10.7 vs controls mean 12.35, 95 % CI 9.2–15.5, pmol uridine/gHb/min; *P* = 0.031) and expression of hENT1 (mean 50.4 %, 95 % CI 38.1–62.7 %, arbitrary units *n* = 11 vs controls mean 95.23 %, 95 % CI 88.38–102.1 % arbitrary units, *n* = 8; *P* < 0.001) were significantly lower; expression of GLUT-1 (mean 106.9 %, vs control mean 99.75 %; *P* = 0.308) and Band 3 polypeptides (mean 100.1 %, vs control mean 102.84 %; *P* = 0.329) were unchanged.

**Conclusions:**

Nucleoside transporter activity in human erythrocytes sustains intracellular purine nucleotide levels and assists in control of plasma adenosine levels; decreased hENT1 expression and activity in G6PD-deficiency could affect red metabolism and influence a wide spectrum of responses mediated by adenosine receptors.

## Background

Glucose-6-phosphate dehydrogenase (G6PD) deficiency is one of the most common inherited metabolic disorders in humans with highest frequencies found in African, South Asian, Middle Eastern and Mediterranean populations [1, 2]. Over 140 different mutations leading to G6PD deficiency have been reported, most of which are single base changes leading to amino acid substitutions [3, 4]. The WHO has grouped G6PD variants into classes (Classes I to V) based upon residual enzyme activity; each class incorporates a number of genotypes and this genetic variation can account for much of the variation in individual clinical presentation of subjects within a single phenotypic class. Association of high population frequencies of G6PD deficiency with geographical distribution of areas where malaria has been endemic suggested that these genetic variations might confer advantages with respect to resistance to malaria, a conjecture that gave rise to ‘Haldane’s malaria hypothesis’ in which the selective pressures from the disease drive hematological polymorphism in affected populations [5]. This has proved a valuable framework for understanding the presence of a very high frequency of G6PD mutations in different human populations and suggested potential molecular mechanisms for innate resistance of red cells to malaria [6, 7]. The G6PD gene is present on the X chromosome, so phenotypic consequences of genetic variations are readily observed in males. Heterozygous females are genetic mosaics resulting from random X chromosome inactivation (‘Lyonization’) and thus both normal and G6PD-deficient erythrocytes can be observed in their circulation [8, 9]. However, it has not been easy to demonstrate clear heterozygote advantage of G6PD-deficiency in population studies [10, 11].

Glucose-6-phosphate dehydrogenase catalyzes the first and rate-limiting step of the pentose phosphate catabolic pathway which, in mature human erythrocytes, serves as the sole metabolic source of NADPH; this provides the reducing equivalents required to protect the cell from oxidation-induced injury through activity of the glutathione system [12, 13]. Deficiency of G6PD activity renders a red cell highly susceptible to oxidative damage; a clinical consequence of this can be hemolytic anemia. Severity of disease is highly variable and hemolytic crises in G6PD-deficient individuals may be triggered by a variety of oxidative stimuli including certain foods (favism), pharmaceuticals (particularly antimalarial drugs, such as primaquine, and sulphonamide anti-bacterial drugs) and infections [2, 7, 8, 12]. Although all individuals suffering from favism appear to show G6PD-deficiency, not all G6PD-deficient individuals suffer from favism; this has led to speculation that other inherited factor(s) in addition to G6PD genotype differences present within broadly defined phenotypic classes may be important in determining the pathological problems associated with this enzymopathy [14, 15].

The human erythrocyte is unable to synthesize purine nucleotides *de novo* and therefore these cells rely upon exogenous purines, predominantly purine nucleosides, to maintain levels of adenine nucleotides required for red cell energy metabolism [16, 17]. Because of their size and polarity, physiological nucleosides traverse biological membranes very slowly by passive diffusion. Most human cells, including erythrocytes, express nucleoside transporter polypeptides in the plasma membrane to catalyze rapid nucleoside permeation [18]. In the human erythrocyte, rapid transmembrane fluxes of nucleosides are mediated by an equilibrative transporter, hENT1, which shows acute sensitivity towards reversible inhibition by nanomolar concentrations of nitrobenzylthioinosine (often abbreviated to NBMPR or NBTI) and related compounds [19, 20]. Recently it has been reported that hENT1 is also an effective mediator of red cell hypoxanthine transport [21]. The contribution of purine nucleosides (principally inosine secreted by the liver) to erythrocyte metabolism is well established [22, 23] and their significance under different conditions may be explored through numerical analysis of metabolic networks (for example [24]).

Nucleoside transport in erythrocytes is readily inhibited by treatment of cells with diamide [25] suggesting that the transporter is highly sensitive to membrane effects of oxidative stress. Gero and co-workers [26] reported that red cells from patients in Myanmar with G6PD-deficiency showed depressed adenosine uptake, a finding consistent with impaired nucleoside transporter activity. Erythrocytes from Kuwaiti patients diagnosed with beta-thalassemia major, a hemoglobinopathy that imposes oxidative stress in red cells, show decreased nucleoside transport activity and this decrease was associated with a substantial reduction in erythrocyte expression of hENT1 [27]. However, these cells did not show decreased membrane expression of another important nutrient transporter, the equilibrative glucose transporter, GLUT1.

This study investigated hENT1 expression and nucleoside transport activity in erythrocytes with G6PD deficiency to determine if changes in nucleoside transporter expression and activity might be a contributory feature of the variable pathology of erythrocytes demonstrating this common enzymopathy and thus represent a parameter of clinical interest.

## Methods

### Materials

Phosphate buffered saline (PBS) was prepared as 137 mM NaCl, 2.7 mM KCl, 4.3 mM Na_2_HPO_4_, 1.76 mM KH_2_PO_4_, pH 7.4. Electroblotting transfer buffer was prepared as 25 mM Tris, 192 mM glycine pH 8.3, 5 % (v/v) methanol. Radiotracer uridine [5,6-^3^H], 37 Ci/mmol was from MP Biomedical, USA. Protease inhibitors; phenylmethylsulfonyl fluoride (PMSF) and protease inhibitor cocktail; Sigma product P2714, were from Sigma Chemical Co, USA. Dilazep was a generous donation from Hoffman-La Roche Ltd, Canada. Rabbit antiserum directed against GLUT-1 polypeptides (C-terminal epitope) was from Alpha Diagnostics, San Antonio, TX, USA. Murine monoclonal IgG specific for human Band 3 polypeptides (B9277; clone BIII-136 directed against N-terminal cytoplasmic epitope) was from Sigma Chemical Co, USA. Murine monoclonal antibody specific for hENT1 (10D7G2) was a gift from Professor Carol Cass, Department of Oncology, University of Alberta, Canada.

### Subjects and samples

Blood samples (2 ml to 5 ml volumes in heparin or acid citrate dextrose anticoagulant) were obtained with written informed consent, mostly as surplus from samples used for routine hematological monitoring of patients with a laboratory demonstration of G6PD-deficiency. Individuals taking medication(s), or with any indication of recent hemolytic crisis or blood transfusion were excluded from the study; twenty one samples were analyzed. Quantitative in vitro determination of G6PD activity in erythrocytes used RANDOX G-6-PDH kit and LXI725 Beckman Coulter analyzer using values for G6PD deficiency set by the Kuwait Ministry of Health (reference range 70–180 mU per 10^9^ cells); less than 70 mU per 10^9^ cells was considered to be G6PD-deficient. The average value for the study group was 24.47 mU with range from 5.6–51.5 mU (WHO Class III deficiency). Samples were cooled on ice immediately after collection (Sabah or Mubarak Al-Kabeer Hospitals, Kuwait) and processed within 48 h of collection. Control samples were obtained with written informed consent from blood donors attending the Central Blood Bank, Jabriya, Kuwait; control subjects were of Arab ethnicity, mostly of Kuwaiti nationality. Ethical approval for the study was granted by the Ethics Committee, Faculty of Medicine, Kuwait University.

### Processing of blood samples

Blood samples were subjected to centrifugation (500 × g, 5 min) to remove plasma proteins, platelets, and white blood cells. Following centrifugation, the red cell fraction was washed twice (resuspension in PBS and centrifugation, 500 × g, 5 min) to give an erythrocyte preparation depleted of extracellular medium and plasma proteins. For uridine uptake measurements, cell suspensions of about 30 % hematocrit in PBS were prepared. Erythrocyte counts of cell suspensions were performed manually using a hemocytometer (Hycor, Fisher Scientific, UK). Hemoglobin was determined spectroscopically after lysis of red cells in Drabkin reagent; reticulocyte counts were obtained by use of an automated hematological analyzer (Beckman Coulter GEN-S, USA).

### Red cell membrane preparation

Red cell membranes were prepared by a conventional hypotonic cell lysis protocol [27]. Briefly, washed erythrocytes (above) were lysed in 20–50 volumes of ice-cold buffer (5 mM phosphate, pH 8.0) in the presence of 0.1 mM phenylmethylsulfonyl fluoride (PMSF) and a commercial cocktail of protease inhibitors; the cocktail was diluted (50 μl/100 ml) in lysis medium and PMSF added immediately prior to addition of erythrocytes. Membranes were collected by high-speed centrifugation (15 min, ≈ 26,000 × g) and the supernatant fluid discarded. Membranes were washed by repeated (usually 5–6 times) resuspension and centrifugation until an off-white membrane preparation was obtained. Protein concentrations were estimated by the Lowry method [28] in the presence of sodium dodecyl sulfate using bovine serum albumin as a standard. Membranes were stored as small aliquots in 5 mM phosphate, pH 8.0 at −80 °C prior to electrophoretic analysis.

### Assay of uridine transport in human erythrocytes

Uptake of ^3^H-uridine for short time periods at room temperature was measured using a conventional ‘inhibitor-oil stop’ protocol [27]. Briefly, 50 μl portions of silicone oil mixture (density 1.034 g/ml; Dow Corning 550 and 200/1c mixed in a ratio of 86:14) were placed in 1.5 ml microcentrifuge tubes and 50 μl portions of erythrocyte suspension (~30 % hematocrit) introduced as a single drop upon the oil layer. Uridine radiotracer solution in PBS containing ^3^H-uridine radiotracer at a concentration of 2 μCi/ml was prepared. Transport was initiated by rapid addition of uridine tracer solution and terminated after a precise interval by rapid addition of 500 μl ice-cold ‘stopper’ solution (40 μM dilazep in PBS) followed by immediate centrifugation (Eppendorf centrifuge 54215C, full speed ≈ 14,000 × g) for 1 min to generate a red cell pellet separated by a layer of silicone oil from supernatant fluid. Supernatant fluid was carefully removed and the tube above the oil layer washed by careful addition and removal of about 1 ml distilled water. After removal of oil, the erythrocyte pellet was disrupted by addition of 200 μl 0.5 % (v/v) Triton X-100 in distilled water and vortex mixing. Protein was precipitated by addition of 200 μl of 5 % (w/v) trichloroacetic acid and brief vortex mixing and the precipitate pelleted by centrifugation (full speed, 4 min). A 300 μl portion of the supernatant was mixed with 3 ml scintillation fluid (Aquasol-2, Beckman Instruments, USA) and radioactivity determined using a Beckman LS 6000TA scintillation counter. Radioactivity associated with extracellular medium trapped in the erythrocyte pellet was estimated from tubes in which ‘stopper’ solution was added immediately prior to uridine tracer addition or the addition of erythrocyte suspension to a mixture of uridine and stopper solution. Uptake of uridine after 5 s of incubation was determined as the mean of quadruplicate measurements. Hemoglobin concentrations in hemolysates were determined using Drabkin reagent and measurement of absorbance at 540 nm.

### Electrophoretic analysis and immunoblot protocols

Membrane proteins were analyzed by SDS-PAGE (Bio Rad Mini-gel system according to protocols provided by BioRad, USA; 10 % polyacrylamide gel, 4 % stacking gel, Laemmli discontinuous buffer system, 200v) after solubilization of proteins in sample buffer in the presence of β-mercaptoethanol as reported previously [27]. For each analysis, quadruplicate gels were prepared with two being used for SDS-PAGE analysis and two for immunoblots. Proteins were transferred to polyvinylidine difluoride (PVDF) membranes (0.45 micron pore size, Millipore Immobilon-P, Millipore, USA or BioRad, USA) overnight at 30 V in a cold room (6–8 °C) using Towbin transfer buffer with 5 % or 10 % methanol. SDS-PAGE gels were stained with Coomassie blue R-250 (0.25 % w/v in 50 % v/v methanol, 10 % v/v acetic acid) and destained (5 % v/v methanol, 7.5 % v/v acetic acid) prior to scanning densitometry (Syngen Bioimaging Systems).

After electrophoretic transfer of proteins (confirmed by reversible staining with Ponceau S in 1 % acetic acid and washing with PBS), blots were allowed to dry at room temperature. Protocols for antigen detection and quantitation followed those described previously [27] using blocking solutions of 5 % non-fat milk powder dissolved in 0.2 % (v/v) Tween-20 in PBS. Incubations with primary antibodies were at ≈ 6 °C for 6–18 h with gentle agitation; incubations with secondary antibodies were for 2–4 h. Experimental controls from which the primary antibodies were omitted showed no staining in pertinent regions of the blot (data not shown). Proteins were detected by probing blots first with a murine monoclonal antibody specific for hENT1 (antibody 10D7G2; [29]) followed by horseradish peroxidase-conjugated second antibodies and commercial chemiluminescence detection systems (ECL plus, RPN1232 and ECL, RPM 2108 kits, Amersham Biosciences, UK) and Biomax Light Chemiluminescence film (Kodak, USA). Film was developed manually and band densities quantitated using a densitometry analysis system (Syngene Chemi Genius Bio Imaging System). Blots were subsequently stripped (5 mM Tris pH 6.8, 2 % SDS, 100 mM β-mercaptoethanol in deionized H_2_O) at 50 °C for 20 min with agitation at 5-min intervals. Membranes were washed twice for 10 min in PBS at room temperature before blocking and re-probing for GLUT-1 glucose transporter polypeptides. Blots were then subjected to a further round of stripping and blocking to detect and quantify Band 3 (also termed AE1) polypeptides. The level of target protein (hENT1, GLUT1, AE1) was compared to dilutions of a standard membrane preparation (a red cell membrane preparation from a single normal donor stored in small aliquots at −80 °C) present on the same blot. Multiple film exposures were taken from each blot to ensure that for quantitative analysis the densities of unknowns fell within the range of densities of different amounts of standard membrane preparation transferred on the same blot.

### Data analysis

Two identical replicate blots were prepared and analyzed for each membrane sample (from G6PD-deficient or control individuals). Results of replicate blots were averaged and the average values used for subsequent analysis. Tests of distribution curves were performed; these supported application of non-parametric tests to investigate changes in membrane transporter (hENT1) expression between G6PDH-deficient patients and controls. Statistical analysis was performed using SPSS and Graphpad Prism software. *P* values below 0.05 were taken to be significant.

## Results

**Fig. 1:**
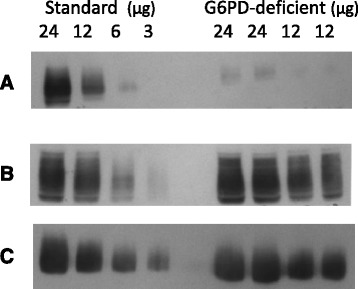
Immunoblot showing protein expression in control and G6PD-deficient erythrocytes: Comparison of erythrocyte membranes from control and G6PD-deficient individuals. Images of an immunoblot probed with antibodies specific for hENT1 (panel **a**) then stripped and re-probed with antibodies directed against GLUT1 polypeptides (panel **b**) before stripping and re-probing for AE-1 (Band 3) polypeptides (panel **c**). Control membrane preparation (24, 12, 6 and 3 μg membrane protein); membranes from G6PDH-deficient individual (duplicate lanes of 24 and 12 μg membrane protein)

**Fig. 2:**
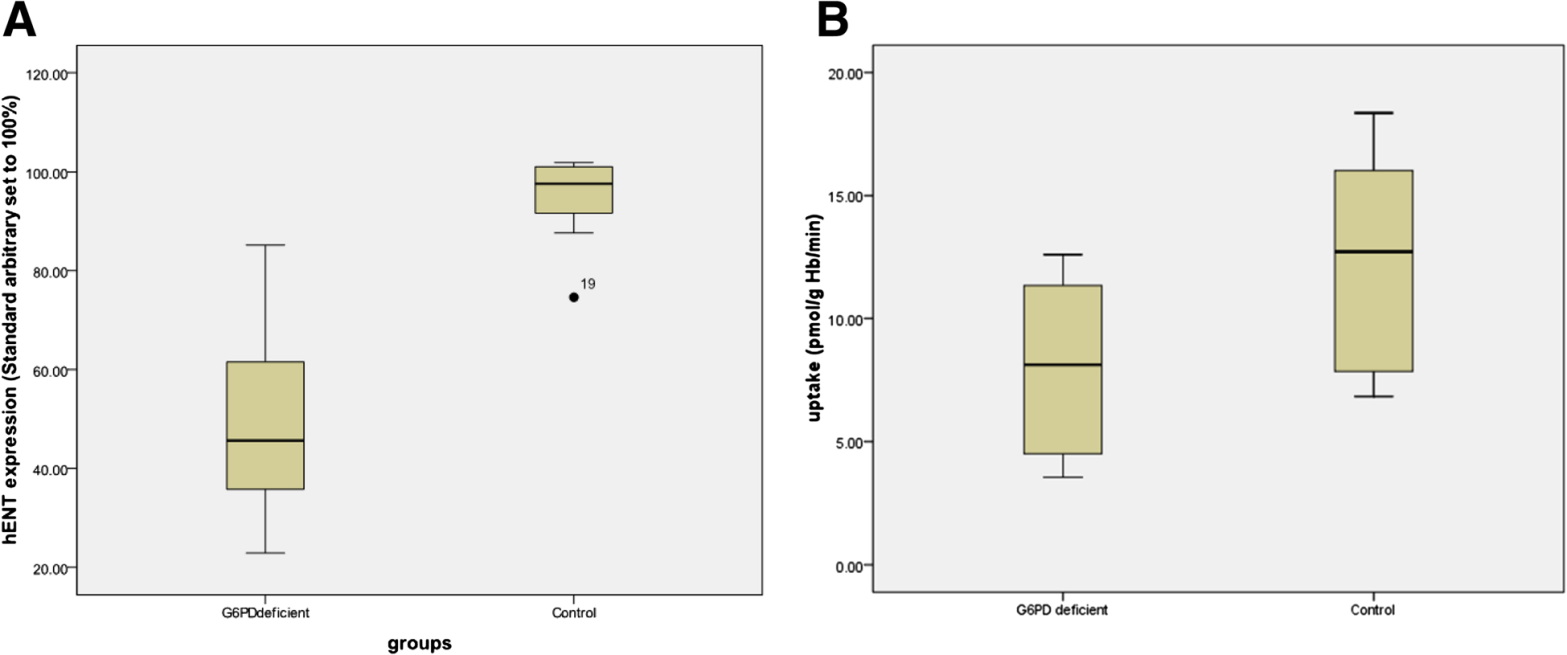
Expression of nucleoside transporter polypeptides and uridine transport rates in G6PD-deficient and control subjects: Expression and activity of nucleoside transporter hENT1 in G6PD-deficient and control subjects. Panel **a** shows hENT-1 protein expression estimated from immunoblots; transporter expression from both G6PDH-deficient patients and control subjects normalized to total membrane protein were compared to a common standard membrane preparation (arbitrarily set to 100 %, vertical axis). Panel **b** shows comparison of initial rates of uridine flux into washed erythrocytes at room temperature

**Fig. 3:**
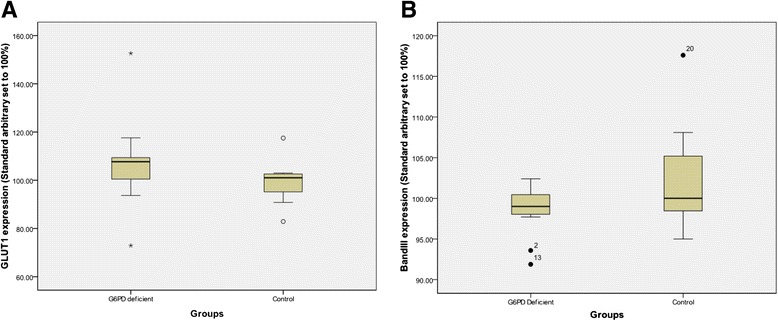
Expression levels of glucose transporter GLUT1 and anion exchanger AE-1 polypeptides in erythrocyte membranes from G6PDH-deficient patients and control subjects: Expression levels of glucose transporter GLUT1 and anion exchanger AE-1 polypeptides in erythrocyte membranes from G6PDH-deficient patients and control subjects. Panel **a** shows levels of GLUT1 glucose transporter and (Panel **b**) anion exchanger AE-1 (Band 3) polypeptides. Transporter polypeptide expression was normalized to total membrane protein and samples from both G6PDH-deficient patients and control subjects were compared to a common standard membrane preparation (arbitrarily set to 100 %, vertical axis)

No consistent qualitative differences in the protein profiles between GD6PD-deficient and control erythrocyte membranes were observed in SDS-PAGE gels stained with Coomassie blue (data not shown). However, decreased levels of hENT1 in G6PD-deficient cells were apparent from immunoblots (Figs. 1 and 2). Decrease in expression of hENT1 was significant (mean 50.4 %, 95 % CI 38.1–62.7 %, *n* = 11 *P* < 0.001 vs controls mean 95.2 %, 95 % CI 88.4–102.1 % *n* = 8; arbitrary units) and was paralleled by a finding of significantly lower uridine uptake in G6PD-deficient cells than in normal erythrocytes (mean 8.18, 95 % CI 5.6–10.7 pmol uridine/g Hb/min compared to mean 12.35, 95 % CI 9.2–15.5 pmol uridine/g Hb/min; *P* = 0.031) Fig. 3. Expression of GLUT-1 (Fig. 3; mean 106.9 %, 95 % CI 96.7–117.1 % *n* = 11 vs control mean 99.8 %, 95 % CI 91.4–108.1 % *n* = 8; arbitrary units) and Band 3 polypeptides (mean 100.1 %, 95 % CI 97.2–102.8 % *n* = 11 vs control mean 102.84 %, 95 % CI 95.72–109.96 % *n* = 8) were not significantly different with *P* values of 0.308 and 0.329, respectively.

## Discussion

This study shows that erythrocytes from individuals with G6PD-deficiency have lower amounts of hENT1 nucleoside transporter polypeptides in their membranes and show lower nucleoside transport activity. This is not a general change in red cell membrane protein composition since the glucose transporter GLUT1 and anion exchanger AE1 polypeptides are not appreciably affected. Decrease in expression of hENT1 showed substantial variation between different individuals with G6PD deficiency. Participants in the study were not genotyped; however, previous reports [30, 31] and review of the patient notes suggest that the majority of our study population would be G6PD Mediterranean. G6PD genotype could be a significant factor for hENT1 expression and thus contribute to the variation observed within our G6PD-deficient group. The current study did not furnish evidence regarding the mechanism(s) responsible for the decrease in hENT1 levels; we would speculate that likely possibilities would include decreased transcriptional efficiency early in the red cell developmental sequence or alteration in protein sorting resulting in vesicular loss of hENT1 along with components such as the transferrin receptor when the membrane is remodeled late in cell maturation. We hope to address this question, and potential role of genotype differences, in future investigations.

In G6PD-deficiency, reduced expression of hENT1 in the erythrocyte will lead to lower transmembrane purine fluxes; these lower fluxes will be further curtailed by decreased transporter activity under conditions of oxidative stress. This would be expected to have effects on red cell metabolism and stability, and on clearance of adenosine from the plasma which could influence local extracellular adenosine concentrations.

The red cell utilizes extracellular purines both to maintain its intracellular purine nucleotide pool and, through the action of purine nucleoside phosphorylase, may exploit the pentose moiety for energy production. It is tempting to speculate that decreased purine transport could be of significance in malaria infection since it has been established that the intraerythrocytic malaria parasite is dependent on host cell purines, and the extracellular milieu, for a supply of purines to support metabolic activity and cellular development [32]. Following invasion, the parasite inserts polypeptides into the host erythrocyte membrane that modify solute permeation; however, even after these changes, it is believed that the parasite still relies upon hENT1 present in the host cell membrane to mediate purine nucleoside entry essential for parasite development [33]. Intracellular oxidative stress in erythrocytes is a general characteristic of the most prominent inherited hemoglobinopathies (thalassemias, sickle cell disease) and enzymopathy (G6PD-deficiency) present in human populations subjected to endemic malaria; in vitro studies have demonstrated that the parasite is intolerant of oxidative stress [34]. As noted previously, nucleoside transport activity of ENT1 in red cells is readily inhibited by oxidant treatment of cells in vitro [25]. It is possible that a combination of oxidative stress and limited purine availability could produce a synergistic effect in slowing parasite growth and replication within the host cell thus increasing the chances of recognition, sequestration and destruction of an infected cell before merozoite release.

We might also surmise that variation in hENT1 expression in G6PD deficiency could produce a spectrum of metabolic constraint that could contribute to differential susceptibility to favism and/or drug sensitivity between different individuals with G6PD-deficiency. This hypothesis predicts a positive association between lower erythrocyte hENT1 expression and likelihood and severity of favism or drug sensitivity in different individuals showing similar G6PD-deficiency phenotypes. Changes to plasma adenosine levels may also influence erythrocyte metabolism and stability as demonstrated in recent investigations of sickle cell disease [35].

## Conclusions

Expression of hENT1 in erythrocytes is significantly decreased in G6PD-deficiency and this deficiency is associated with lower nucleoside permeation rates. Concordance of changes in hENT1 expression and nucleoside transport indicates that in the absence of complicating factors, such as infection or exogenous oxidative stress, the principal cause of changes to nucleoside flux rates is expression of transporter rather than changes to functional properties of the transporter in the plasma membrane.

## Abbreviations

G6PD: glucose 6-phosphate dehydrogenase
Hb: hemoglobin
SDS-PAGE: sodium dodecyl sulfate polyacrylamide gel electrophoresis

## References

[1] Nkhoma ET, Poole C, Vannappagari V, Hall SA, Buetler E (2009). The global prevalence of glucose-6-phosphate dehydrogenase deficiency: a systematic review and meta-analysis. Blood Cells Mol Dis.

[2] Capellini MD, Fiorelli G (2008). Glucose-6-phosphate dehydrogenase deficiency. Lancet.

[3] Beutler E, Vulliamy TJ (2002). Hematologically important mutations: glucose-6-phosphate dehydrogenase. Blood Cells Mol Dis.

[4] Minucci A, Moradkhani K, Hwang MJ, Zuppi C, Giardina B, Capoluongo E (2012). Glucose-6-phosphate dehydrogenase (G6PD) mutations database: review of the "old" and update of the new mutations. Blood Cells Mol Dis.

[5] Haldane JBS (1949). The rate of mutation of human genes. Hereditas Suppl.

[6] López C, Saravia C, Gomez A, Hoebeke J, Patarroyo MA (2010). Mechanisms of genetically-based resistance to malaria. Gene.

[7] Mason PJ, Bautista JM, Gilsanz F (2007). G6PD deficiency: the genotype-phenotype association. Blood Rev.

[8] Beutler E (1994). G6PD deficiency. Blood.

[9] Peters AL, Van Noorden CJ (2009). Glucose-6-phosphate dehydrogenase deficiency and malaria: cytochemical detection of heterozygous G6PD deficiency in women. J Histochem Cytochem.

[10] Hedrick P (2011). Population genetics of malaria resistance in humans. Heredity.

[11] Luzzatto L (2012). G6PD deficiency and malaria selection. Heredity (Edinb).

[12] Jollow DJ, McMillan DC (2001). Oxidative stress, glucose-6-phosphate dehydrogenase and the red cell. Adv Exp Med Biol.

[13] Stanton RC (2012). Glucose-6-phosphate dehydrogenase, NADPH, and cell survival. IUBMB Life.

[14] Stamatoyannopoulos G, Fraser GR, Motulsky AC, Fessas P, Akrivakis A, Papayannopoulou T (1966). On the familial predisposition to favism. Am J Hum Genet.

[15] Maren C, Repetto L, Forteloni G, Meloni T, Gaetani GF (1984). Favism: looking for an autosomal gene associated with glucose-6-phosphate dehydrogenase deficiency. J Med Genet.

[16] Parks RE, Crabtree GW, Kong CW, Agarwal RP, Agarwal KC, Scholar EM (1975). Incorporation of analog purine nucleosides into the formed elements of human blood: erythrocytes, platelets and lymphocytes. Ann N Y Acad Sci.

[17] Dudzinska W, Lubkowska A, Dolegowska B, Safranow K, Jakubowska K (2010). Adenine, guanine and pyridine nucleotides in blood during physical exercise and restitution in healthy subjects. Eur J Appl Physiol.

[18] King AE, Ackley MA, Cass CE, Young JD, Baldwin SA (2006). Nucleoside transporters: from scavengers to novel therapeutic targets. Trends Pharmacol Sci.

[19] Baldwin SA, Beal PR, Yao SY, King AE, Cass CE, Young JD (2004). The equilibrative nucleoside transporter family, SLC29. Pflugers Arch.

[20] Young JD, Yao SYM, Sun I, Cass CE, Baldwin SA (2008). Human equilibrative nucleoside transporter (ENT) family of nucleoside and nucleobase transporter proteins. Xenobiotica.

[21] Yao SY, Ng AM, Cass CE, Baldwin SA, Young JD (2011). Nucleobase transport by human equilibrative nucleoside transporter 1 (hENT1). J Biol Chem.

[22] Zeidler RB, Metzler MH, Moran JB, Kim HD (1985). The liver is an organ site for the release of inosine metabolized by non-glycolytic pig red cells. Biochim Biophys Acta.

[23] Young J, Paterson AR, Henderson JF (1985). Nucleoside transport and metabolism in erythrocytes from the Yucatan miniature pig. Evidence that inosine functions as an in vivo energy substrate. Biochim Biophys Acta.

[24] Wiback SJ, Palsson BO (2002). Extreme pathway analysis of human red blood cell metabolism. Biophys J.

[25] Gero AM, Wood AM, Hogue DL, Upston JM (1991). Effect of diamide on nucleoside transport in Plasmodium falciparum and Babesia bovis infected erythrocytes. Mol Biochem Parasitol.

[26] Myint-Oo O'SWJ, Gero AM (1997). Laboratory and field comparisons of adenosine influx in Plasmodium falciparum and Plasmodium vivax infected erythrocytes with genetic abnormalities from patients in Myanmar. Southeast Asian J Trop Med Public Health.

[27] Al-Massaeid AL, Craik JD (2009). Decreased nucleoside transport and hENT1 transporter expression in beta-thalassemia major. Med Princ Pract.

[28] Lowry OH, Rosebrough NJ, Farr AL, Randall RJ (1951). Protein measurement with the Folin phenol reagent. J Biol Chem.

[29] Jennings LL, Hao C, Cabrita MA, Vickers MF, Baldwin SA, Young JD (2001). Distinct regional distribution of human equilibrative nucleoside transporter proteins 1 and 2 (hENT1 and hENT2) in the central nervous system. Neuropharmacology.

[30] Samilchuk E, D'Souza B, Al-Awadi S (1999). Population study of common glucose-6-phosphate dehydrogenase mutations in Kuwait. Hum Hered.

[31] Alfadhli S, Kaaba S, Elshafey A, Salim M, AlAwadi A, Bastaki L (2005). Molecular characterization of glucose-6-phosphate dehydrogenase gene defect in the Kuwaiti population. Arch Pathol Lab Med.

[32] Cassera MB, Zhang Y, Hazleton KZ, Schramm VL (2011). Purine and pyrimidine pathways as targets in Plasmodium falciparum. Curr Top Med Chem.

[33] Quashie NB, Ranford-Cartwright LC, de Koning HP (2010). Uptake of purines in Plasmodium falciparum-infected human erythrocytes is mostly mediated by the human equilibrative nucleoside transporter and the human facilitative nucleobase transporter. Malar J.

[34] Preuss J, Jortzik E, Becker K (2012). Glucose-6-phosphate metabolism in Plasmodium falciparum. IUBMB Life.

[35] Zhang Y, Dai Y, Wen J, Zhang W, Grenz A, Sun H (2011). Detrimental effects of adenosine signaling in sickle cell disease. Nat Med.

